# AI-assisted computed tomography analysis for pre-procedural planning prior to TAVI

**DOI:** 10.1007/s00392-025-02790-6

**Published:** 2025-11-12

**Authors:** Mani Arsalan, Hanna Schneider, Kerstin Piayda, Philipp Christian Seppelt, Arnaud Van Linden, Stephan Fichtlscherer, Florian Hecker, David Leistner, Thomas Walther

**Affiliations:** 1 Department of Cardiac Surgery, University Hospital of the Goethe University, Theodor-Stern-Kai 7, 60590 Frankfurt, Germany; 2 Department of Cardiology and Angiology, Medical Clinic I, University Hospital of the Justus Liebig University, Klinikstrasse 33, 35392 Giessen, Germany; 3 Department of Cardiology & Angiology, Medical Clinic III, University Hospital of the Goethe University, Theodor-Stern-Kai 7, 60590 Frankfurt, Germany; 4 Department of Cardiothoracic and Thoracic Vascular Surgery, University Hospital Würzburg, Würzburg, Germany; 5 Department of Cardiology, Heart Center Segeberger Kliniken GmbH, Bad Segeberg, Germany

**Keywords:** Artificial intelligence, Deep-learning, Computed tomography, Aortic valve, Transcatheter aortic valve implantation

## Abstract

**Background:**

Several software programs have specifically been developed to analyse cardiac computed tomography prior to transcatheter aortic valve implantation (TAVI). However, they are not able to perform a complete analysis independently. We report the performance of a fully automated, deep learning-based algorithm for pre-procedural CT analysis as compared to the current clinical standard.

**Methods:**

Patients with symptomatic severe aortic stenosis undergoing TAVI were retrospectively enrolled. The pre-procedural dataset was analysed by both a standard TAVI CT-analysis software and by a fully automated CT analysis platform with a deep learning-based algorithm.

**Results:**

Ninety-eight patients were included in the analysis. The mean annulus diameter was 24.4 ± 2.4 mm (conventional = 3mensio, Pie Medical Imaging, 3 M) vs. 24.0 ± 2.4 mm (artificial intelligence = AI), mean absolute error (MAE): 0.64 mm, mean absolute percentage error (MAPE): 2.6%. The mean annulus perimeter was measured at 77.7 ± 7.4 mm (3 M) vs. 76.1 ± 7.5 mm (AI), MAE: 2.26 mm, MAPE: 2.9%. The mean annulus area was calculated at 468.9 ± 92.1 mm^2^ (3 M) vs. 455.6 ± 91.0 mm^2^ (AI), MAE: 22.4 mm^2^, MAPE: 4.8%. The intraclass correlation coefficients (ICCs) of all abovementioned parameter were > 0.95 showing an excellent correlation between the two methods. The distance from the annulus to the left coronary artery depicted to 14.0 ± 3.2 mm (3 M) vs. 12.6 ± 2.8 mm (AI), MAE: 2.1 mm, MAPE: 14.3%. The distance to the right coronary artery was 17.1 ± 2.7 mm (3 M) vs. 16.5 ± 3.2 mm (AI), MAE: 1.7 mm, MAPE: 10.1%. The ICCs of the distances to the coronary ostia showed good correlation between both methods.

**Conclusion:**

In this retrospective analysis, a deep learning-based analysis of pre-procedural CT datasets showed good to excellent correlation with conventional assessment for the preprocedural TAVI CT measurements. AI-based fully automated CT analysis could emerge to a valuable alternative to conventional CT assessment in the pre-procedural-planning for TAVI.

**Graphical Abstract:**

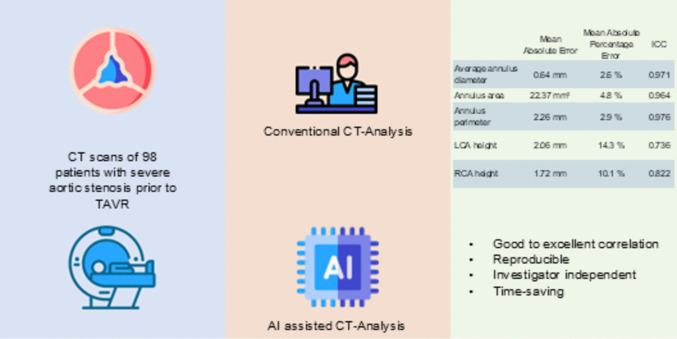

**Supplementary Information:**

The online version contains supplementary material available at 10.1007/s00392-025-02790-6.

## Introduction

Over the last decade, transcatheter aortic valve implantation (TAVI) has emerged to the gold standard for treating elderly patients with symptomatic severe aortic stenosis (AS). Pre-procedural cardiac computed tomography (CT) was discovered early on as an essential tool to optimize implantation and clinical results [1]. It assists with case-specific valve selection and sizing and provides valuable information on access site anatomy and morphology.

For this reason, specific post-processing CT software was developed to simplify TAVI-CT analysis. Amongst others, 3mensio software (Pie Medical Imaging BV, Netherlands) is a widely used software for procedural TAVI planning. Early on, the post-processing software generated CT-analysis with high reproducibility, even for inexperienced users. It also reduces the time required for analysis and has a better predictive value for complications such as post-procedural aortic regurgitation as compared to manual measurement [2, 3]. However, inter-observer agreement and reproducibility depends on the observer’s experience [4]. Automatic measurements have long been desired but have not shown reliable results without manual corrections [5]. Thus, semi-automatic software seems to provide the best measuring results, they come along with a certain time effort.

In recent years, artificial intelligence (AI) and deep learning (DL) have revolutionized various fields, including scientific research. One area where AI has shown immense promise is in the realm of measurements. Thus, due to AI and DL, complete automatic analysis of pre-procedural CT datasets could be possible in the meantime. This would address the aforementioned shortcomings and could lead to a higher level of accuracy, reduce workforce burden, and enable rapid and comprehensive CT analysis within seconds. Hence, we compared a fully automated deep learning-based CT analysis with the current standard CT post-processing software.

## Methods

- The aim of the study was to evaluate the agreement between the AI platform and 3mensio for key anatomical measurements (annulus diameter, perimeter, area, and coronary ostia distances), as assessed by ICC, MAE, and MAPE. Secondary outcomes included analysis time and reproducibility. All patients with symptomatic severe aortic stenosis who underwent pre-procedural CT imaging for TAVI within the defined study period were screened. Exclusion criteria included degenerated surgical valves, incomplete imaging datasets, and poor image quality precluding accurate analysis. The final cohort consisted of all patients fulfilling these criteria. For each patient, CT datasets were analysed in two independent ways: conventionally using the 3mensio Structural Heart software, version 10.1 (3 M, Pie Medical Imaging BV, Netherlands) by one of three experienced operators who at least performed 200 TAVI-CT analyses independently
- Fully automatic by an AI platform (AI, Laralab GmbH, Munich, Germany)

For the conventional approach, after automatic segmentation of the ascending aorta and stretching of the bordering vessel was done by the software, the user manually adjusts the lumen centreline and defines the annulus plane by marking the nadirs of the leaflets. Afterwards, measurements of the average diameter, perimeter, and area of the aortic annulus, as well as the distance to the coronary ostia were manually performed. The exact work-flow is described elsewhere [2]. All measurements were performed semiautomatically (version 10.1) with manual correction permitted as required. Deep learning-based segmentation was not used. To evaluate differences between different users of the conventional platform, measurements were repeated by an experienced operator and analysis time was assessed.

The AI platform is a cloud-based software platform that has been trained on 811 CT datasets and 250.198 image slices. The convolutional neural network (CNN) models were trained based on multi-centre data derived from routine clinical practice across multiple institutions originating from different geographical locations, were acquired using different CT scanners, contain a wide range of CT quality images (e.g. high-resolution/no contrast) and indications (e.g. aortic, mitral, tricuspid) and come from both male and female individuals. During training, approximately 80–90% of the data were used for model training and 10–20% for internal validation, ensuring independent evaluation during development. Additionally, it is tested on an extensive dataset that has been separated from the dataset available during development (thus not even available for validation within the training process). Strict quality processes are in place to ensure separation of datasets. For external validation, the performance was tested on datasets in clinical collaborations.

**Fig. 1:**
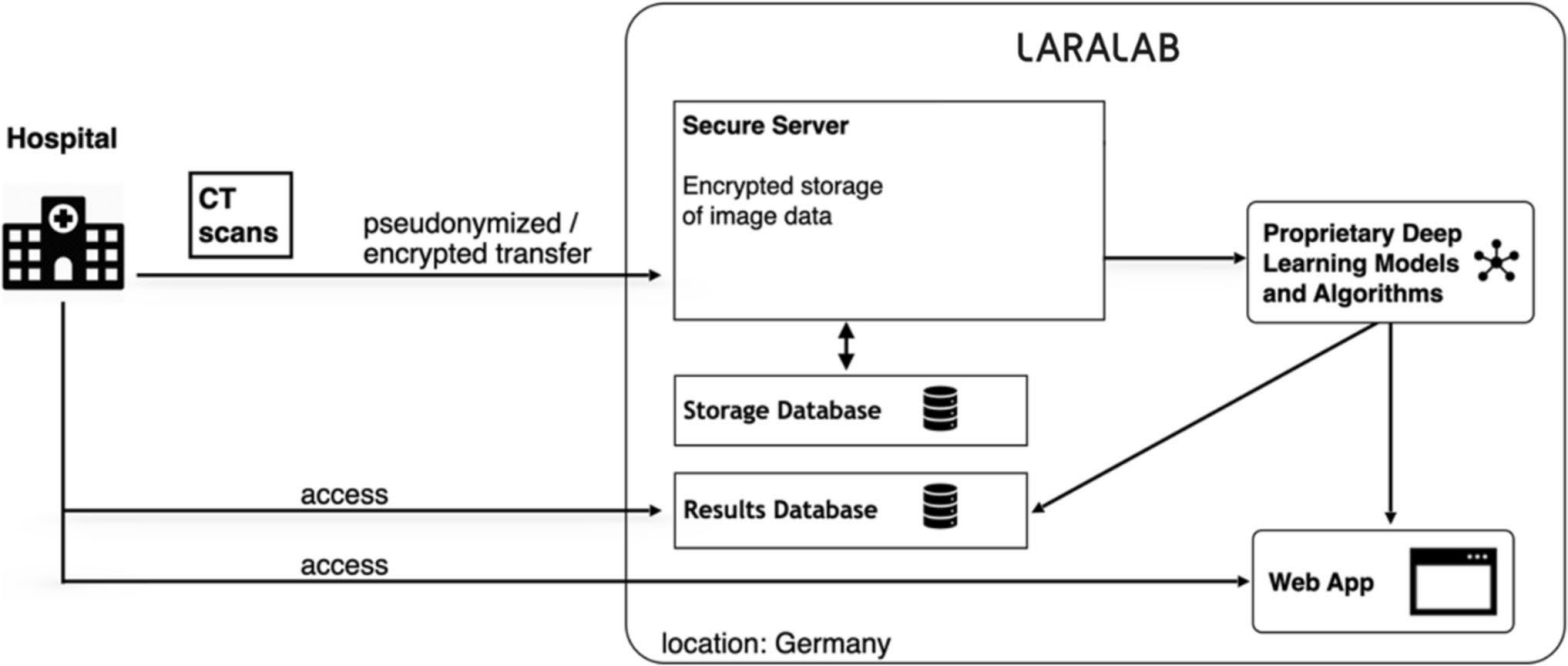
Data management of the artificial intelligence-based platform (transfer, storage and processing)

Corresponding Digital Imaging and Communications in Medicine (DICOM) files are pseudonymized prior to transfer (Fig. 1). The fully automatic segmentation algorithms are based on highly specialised deep learning techniques using CNN, with different networks focusing on different groups of heart anatomies. 3D-models of cardiac anatomy are produced from the binary masks of the segmentations. Characteristic heart planes for multiplanar reconstruction views are calculated based on the 3D-models, and custom algorithms are used to automatically derive a range of measurements based on specific segments.

For example, the AI-driven CT analysis platform identifies the nadirs by a 3D CNN specialised in segmenting aortic cusps and uses these nadirs to determine the annular plane. Another specialised CNN is used to segment the aortic root. Its outer contour is then used to derive the aortic annulus in the annular plane.

The AI model was trained and validated on CT datasets with a wide range of contrast, including low-contrast scans and datasets with non-contrasted regions (e.g. left or right heart). CT datasets with a slice thickness above 3.0 mm or with a significant number of missing slices were automatically rejected. Within the supported range, the model provides robust measurements even for lower-quality datasets. All automated analyses generate a report with dedicated multiplanar reconstruction (MPR) views and, optionally, an interactive planning environment to allow review of the results. In the current beta version, the AI platform is only able to measure the annulus diameter, area and perimeter, as well as the distances from the annular plane to the coronary arteries. Thus, the comparison of 3 M and AI-based measurements were limited to aforementioned parameters.

All CT scans in our study were performed using a Siemens Somatom Force dual source CT scanner. Images were acquired with a slice thickness of 0.3 mm. ECG-gating was used, and end-systolic images were used for further analyses.

The study protocol complies with the Declaration of Helsinki and data collection was approved by the institutional ethics committee (reference number 20–1061) with waiver of individual consent, with a waiver of individual informed consent due to the retrospective and anonymized nature of the data.

### Statistical analysis

Continuous variables were assessed for normal distribution with the Shapiro-Wilks test. Comparison of the differences in annular dimensions and coronary distances were analysed with the paired *t*-test. Concordance between the two methods derived measurements was evaluated using Bland–Altman analysis. In addition, interobserver agreement was assessed by calculating the intraclass correlation coefficients (ICCs), with good agreement defined as ICC > 0.75 and excellent agreement defined as an ICC > 0.9 [6]. Mean absolute error (MAE) and mean absolute percentage error (MAPE) were calculated for all measurements analysing differences between manual measurements and between conventional and AI-based measurements. Data were analysed using SPSS 27 (IBM Corp., Armonk, NY, USA).

## Results

**Fig. 2:**
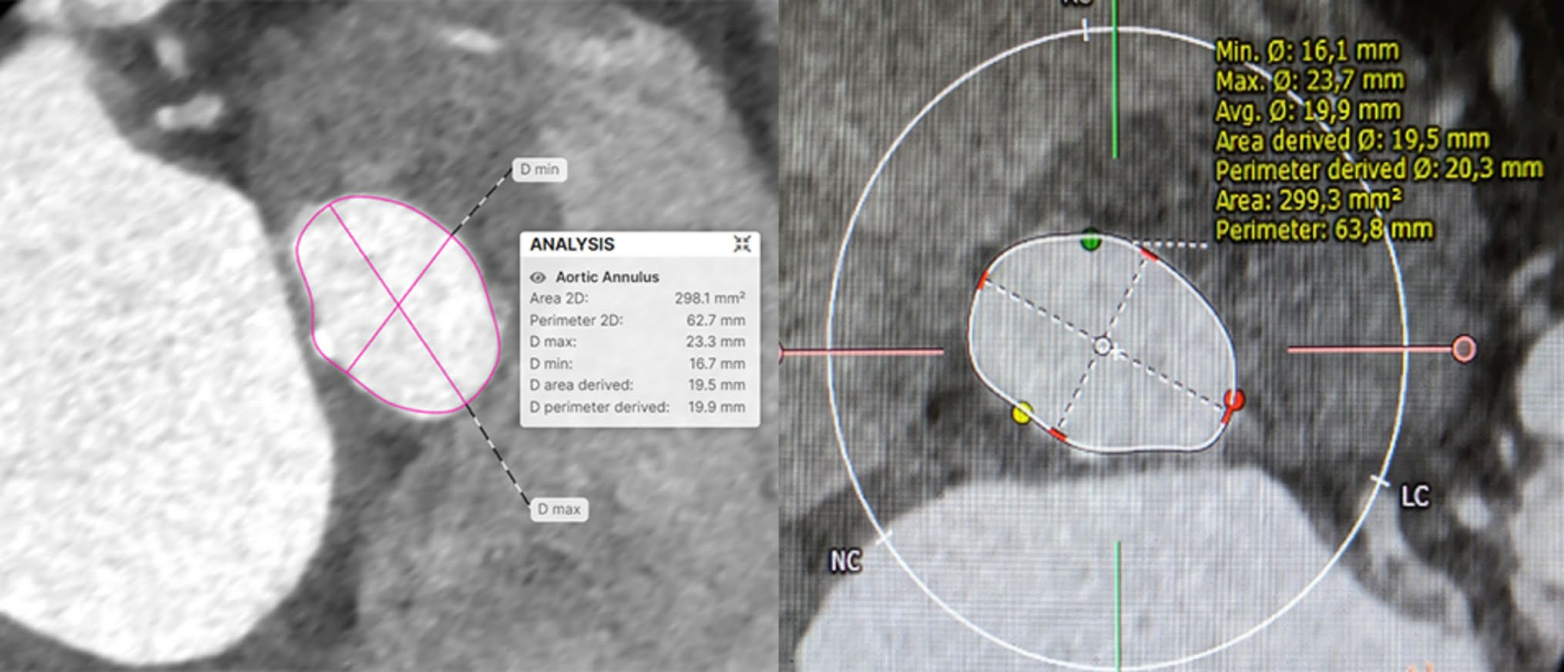
Measurement of the aortic annulus by the artificial intelligence-based platform (left picture), Measurement of the aortic annulus by conventional semi-automatic post-processing CT software (right picture)

Ninety-eight patients were included in the analysis. The mean age of the study population was 80 years, and 42% were female. A comparison of semi-automatic (3 M) and fully automated measurements (AI) of aortic annulus dimensions is presented in Table 1 (Fig. 2). Analysis time for the conventional measurement was 240.8 ± 25.1 s. The AI software analysis of a single-phase CT scan took 180.7 ± 12.4 s.

**Table 1:** Comparison of aortic root anatomy manual and automated measurements (*n* = 98)

	3mensio (SD)	3mensio (95% CI)	AI platform (SD)	AI platform (95% CI)	*p*-value	Interclass correlation coefficient (IQR)
Average annulus diameter (mm)	24.4 ± 2.4	23.9–24.9	24.0 ± 2.4	23.5–24.5	< 0.001	0.971 (0.937; 0.985)
Annulus perimeter (mm)	77.7 ± 7.4	76.2–79.2	76.1 ± 7.5	74.6–77.6	< 0.001	0.964 (0.884; 0.983)
Annulus area (mm^2^)	468.9 ± 92.1	450.4–487.4	455.6 ± 91.0	437.4–473.9	< 0.001	0.976 (0.947; 0.987)
Left coronary artery height (mm)	14.0 ± 3.2	13.4–14.7	12.6 ± 2.8	12.0–13.2	< 0.001	0.736 (0.483; 0.850)
Right coronary artery height (mm)	17.1 ± 2.7	16.6–17.7	16.5 ± 3.2	15.9–17.2	0.013	0.822 (0.730; 0.882)

The measurements differed significantly between both methods, but the numerical differences were small.

**Fig. 3:**
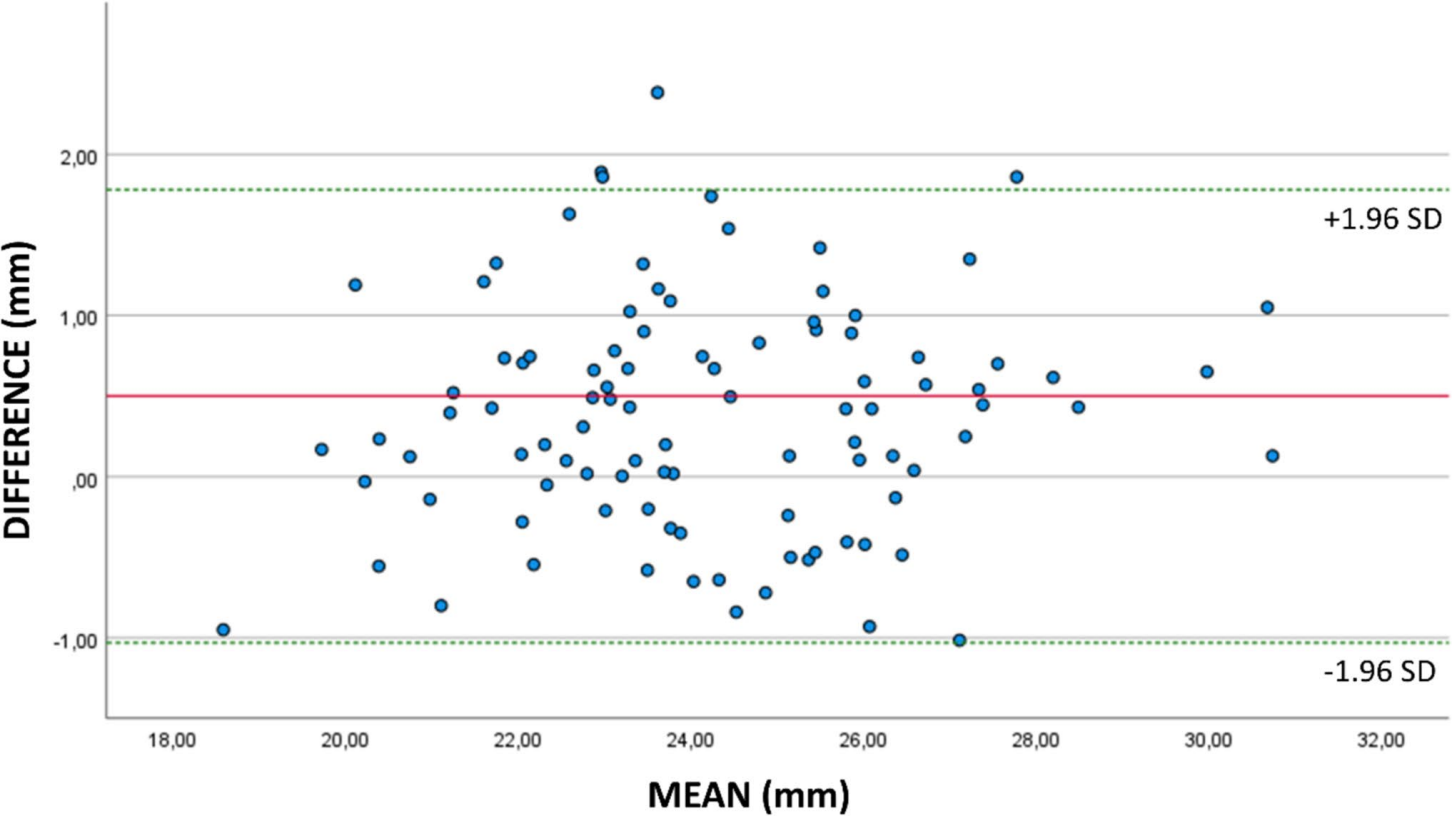
Bland-Altman plot of agreement between both measurement methods for the aortic annulus diameter. The red line indicates the mean difference and the dashed green lines the upper and lower limits of agreement.

The mean aortic annulus diameter was 24.4 ± 2.4 mm (3 M) vs. 24.0 ± 2.4 mm (AI) with a mean of differences of 0.38 ± 0.72 mm (Bland-Altmann plot, Fig. 3). The ICC showed an excellent level of agreement between 3mensio and deep learning-derived measurements 0.971 (0.937; 0.985).

The mean annulus perimeter derived to 77.7 ± 7.4 mm (3 M) vs. 76.1 ± 7.5 mm (AI) with a mean of differences of 1.6 ± 2.33 mm (Bland-Altmann plot, Supplemental Fig. 1) and excellent correlation between both methods ICC: 0.964 (0.884; 0.983).

The mean annulus area was measured as 468.9 ± 92.1 mm^2^ (3 M) vs. 455.6 ± 91.0 mm (AI), with a mean of differences of 13.3 ± 25.2 mm^2^ (Bland-Altmann plot, Supplemental Fig. 2), resulting in an excellent correlation between both methods ICC: 0.976 (0.947; 0.987).

The mean distance from the annular plane to the left coronary artery was determined to be 14.0 ± 3.2 mm (3 M) vs. 12.6 ± 2.8 mm (AI), with a mean of differences of 1.45 ± 2.52 mm (Bland-Altmann plot, Supplemental Fig. 3), and good correlation between both methods ICC: 0.736 (IQR 0.483; 0.850).

The mean distance from the annular plane to the right coronary artery was 17.1 ± 2.7 mm (3 M) vs. 16.5 ± 3.2 mm (AI), with a mean of differences of 0.57 ± 2.2 mm (Bland-Altmann plot, Supplemental Fig. 4), and good correlation between the two methods ICC: 0.822 (0.730; 0.882).

MAE and MAPE between different users of the conventional software were low and comparable to those between 3 M and AI measurements. This confirms the high measurement accuracy of the AI platform (Table 2).

**Table 2:** Mean absolute error (MAE) and mean absolute percentage error (MAPE) between different users of the conventional software (3 M vs. 3 M) and between conventional software and AI platform (3 M vs. AI)

	MAE 3 M vs. 3 M	MAPE 3 M vs. 3 M	MAE 3 M vs. AI	MAPE 3 M vs. AI
Average annulus diameter	0.858 mm	3.5%	0.642 mm	2.6%
Annulus perimeter	1.907 mm	2.5%	2.263 mm	2.9%
Annulus area	31.164 mm^2^	6.8%	22.372 mm^2^	4.8%
Left coronary artery height	1.106 mm	8.3%	2.056 mm	14.3%
Right coronary artery height	1.188 mm	7.1%	1.724 mm	10.1%

## Discussion

This study provides a detailed comparison of preprocedural TAVI-CT analysis between an automatic, deep learning-based software and a conventional semi-automated CT software.

The differences between both methods were small and comparable to the intra- and interindividual variability differences in standard CT-dataset analysis. The prototype of this fully automatic software was able to independently recognize aortic annulus dimensions with an excellent agreement of results to the gold standard and high accuracy. As the algorithm of the AI platform is deterministic, every analysis results in the same measurements and only differs if the algorithm is re-trained.

Semi-automated software solutions for analysing pre-procedural CT datasets were developed early on to facilitate pre-procedural planning. The 3mensio software, as used in this context, showed excellent correlation with complete manual measurements [7], and the results have excellent reproducibility [2, 8].

Several TAVI-specific CT analysis programs are currently available [3, 9, 10]. Horehledova and colleagues confirmed an excellent correlation between manual and semi-automatic measurements using another dedicated post-processing software (Syngo.via, Siemens Healthcare GmbH, Germany) [9]. They could also prove that semi-automatic assessment was significantly faster as compared to manual measurements. The TAVI Analysis software (GE Healthcare, Chicago, IL) showed excellent correlation with manual measurements of the aortic annulus area (ICC 0.91) and perimeter (ICC 0.86) and good correlation regarding annulus diameters (ICC 0.75–0.76) and distance from annulus plane and coronary ostia (ICC 0.75–0.79) [3]. Kocka et al. compared manual measurements to the Philips HeartNavigator software (Philips Healthcare, The Netherlands) [10]. Similar to our study, measurements obtained with the HeartNavigator software were fully automated, and no corrections by users were allowed. Measurements between both methods differed significantly but were numerically small in regards of the aortic annulus perimeter and area. Unfortunately, the study did not perform a correlation analysis.

Meyer and colleagues compared the HeartNavigator software to 3mensio and showed statistically significant higher values for aortic annulus area and perimeter using the fully automatic approach [11]. Consequently, these larger annulus measurements would have led to a larger valve size selection in 13% of the case if the HeartNavigator-based annulus area and perimeter were used to determine the appropriate prosthesis size. Interestingly, Kocka et al. did a similar analysis and showed that in accordance with the manufacturer’s sizing table in 4% of the cases a smaller prothesis and in 16% a larger self-expanding prothesis would have been chosen when using the same software as Meyer et al. [10].

The inter-observer variability of 3 M in the present study is similar to prior reports [8], and the detected differences between 3 M and AI measurements showed to be comparable to these. The differences between both methods might be caused by the suboptimal automatic detection of anatomic structures. While the software allows manual correction, we decided to use the automatically collected measurements. In depth look at the outliers of the Bland–Altman analysis showed that these cases had either poor CT scan quality or extensive calcifications. In clinical routine, valid measurements would be easily obtained by manual correction.

Exact measurement of the aortic annulus is of utmost importance for the correct TAVI prothesis sizing. For this purpose, the aortic annulus area is typically used for sizing balloon-expandable valves and the annulus perimeter for self-expandable prostheses [12]. It is therefore reassuring to note that the correlation between semi-automated and fully automated measurements in the present study showed an excellent correlation for these parameters.

While accurate measurements are important for clinical outcomes, the time required for analysis affects daily clinical practice. In the present study, manual measurements of all parameters that can currently be automatically derived by the AI platform required approximately 4 min per dataset, whereas the fully automated AI analysis required approximately 3 min in total, including data upload and processing time. However, only a very small part of these 3 min is needed by the algorithm for the actual measurements. It is therefore not expected that adding measurements of further parameters will significantly increase the time required, whereas manual measurements using the conventional software are more time-consuming. Furthermore, while the AI platform performs the analysis, no user interaction is required. This time could e.g. be used to upload further CT datasets to the AI platform with a simple mouse click, or pursue other activities.

It is difficult to compare studies investigating the time period spend on TAVI CT analysis. Most studies compare either automatic or semi-automatic analysis with the manual approach. However, the reported analysis time for measurements varied between 3.1 and 17.8 min for the manual [3, 9, 10] and between 1.5 and 3.5 min for the semi-automatic approach.

However, none of these analyses included assessment of the vascular access site and only some assessed the optimal C-arm angulation, which both are important features for procedural planning. In our experience, a realistic time frame for a complete CT-analysis ranges between 8 and 10 min. This is likely to be significantly reduced by the software under review, as the CT analysis takes place in the cloud and does not need human assistance. Although the time savings may appear minimal, they are clinically relevant given the growing number of procedures performed and the reduced reliance on highly specialised personnel. Future iterations of the software might not only be time-saving in daily practice but could potentially suggest valve selection. This recommendation could be based on the analysis of CT data (e.g. vascular access, calcification patterns, annular dimensions, membranous septum-length) and clinical data (e.g. age, existing left bundle branch block, need for percutaneous coronary intervention after TAVI).

This study has several limitations. First, as the fully automatic software is still under development, and we could only compare the parameters which can currently be measured automatically. Second, this pilot study was conducted to evaluate the potential of the software. Therefore, no a priori sample size calculation was performed. While we did not exclude bicuspid and heavily calcified valves, the small number of patients (4 bicuspid valves and 9 severely calcified valves) did not allow to perform a subanalysis of these particular challenging anatomies. However, the AI platform was trained on a broad spectrum of CT datasets including these characteristics.

The current study evaluated the performance of the AI algorithm in a fully automated manner, without manual corrections, to objectively assess algorithm robustness. However, the effect of manual correction on the clinical application should also be investigated in the future. Finally, due to the retrospective nature of the study, we did not evaluate the potential clinical implications in terms of valve selection. However, the Bland–Altman analysis illustrate consistent differences across all aortic annulus sizes and may lead to similar sizing. Given the limited number of patients in our study, we feel that any estimate regarding the proportion of cases in which measurement discrepancies would have led to a different prosthesis size selection would not be reliable. However, as the results are based on a single-centre dataset, they may not be fully generalisable to other populations or imaging protocols. This highlights the importance of a future multi-centre study to investigate the value of the AI platform in clinical practice.

## Conclusion

This retrospective study demonstrates the ability of an AI-based software platform to automatically analyse pre-TAVI CT images, with excellent correlation to the current gold standard semi-automated approach, particularly in the parameter dimensions required for proper prosthesis sizing. However, due to the single-centre, retrospective design and limited sample size, our findings should be considered preliminary. Further development should include reliable measurements for remaining crucial parameters such as the left ventricular outflow tract diameter and assessment of the optimal C-arm angulation for valve deployment and vascular access. Additionally, further multi-centre studies with larger cohorts and external validation are required to determine the clinical impact, generalizability, and potential advantages of AI-assisted CT analysis in routine TAVI planning.

## Supplementary Information

Below is the link to the electronic supplementary material.

ESM 1 Supplementary Material 1. Bland–Altman plot of agreement between both measurement methods for the aortic annulus perimeter. The red line indicates the mean difference and the dashed green lines the upper and lower limits of agreement (PNG 188 KB)

High Resolution Image (TIF 171 KB)

ESM 2 Supplementary Material 2. Bland–Altman plot of agreement between both measurement methods for the aortic annulus area. The red line indicates the mean difference and the dashed green lines the upper and lower limits of agreement (PNG 202 KB )

High Resolution Image (TIF 176 KB)

ESM 3 Supplementary Material 3. Bland–Altman plot of agreement between both measurement methods for the distance between the annular plane and the ostium of the left coronary artery. The red line indicates the mean difference and the dashed green lines the upper and lower limits of agreement (PNG 179 KB)

High Resolution Image (TIF 165 KB)

ESM 4 Supplementary Material 4. Bland–Altman plot of agreement between both measurement methods for the distance between the annular plane and the ostium of the right coronary artery. The red line indicates the mean difference and the dashed green lines the upper and lower limits of agreement (PNG 181 KB )

High Resolution Image (TIF 166 KB)
