# Supplementary figures and images for: AI-assisted computed tomography analysis for pre-procedural planning prior to TAVI
Source: Clin Res Cardiol. 2025 Nov 12;115(7):1199–206. doi: 10.1007/s00392-025-02790-6 (PMC13249624; doi:10.1007/s00392-025-02790-6)

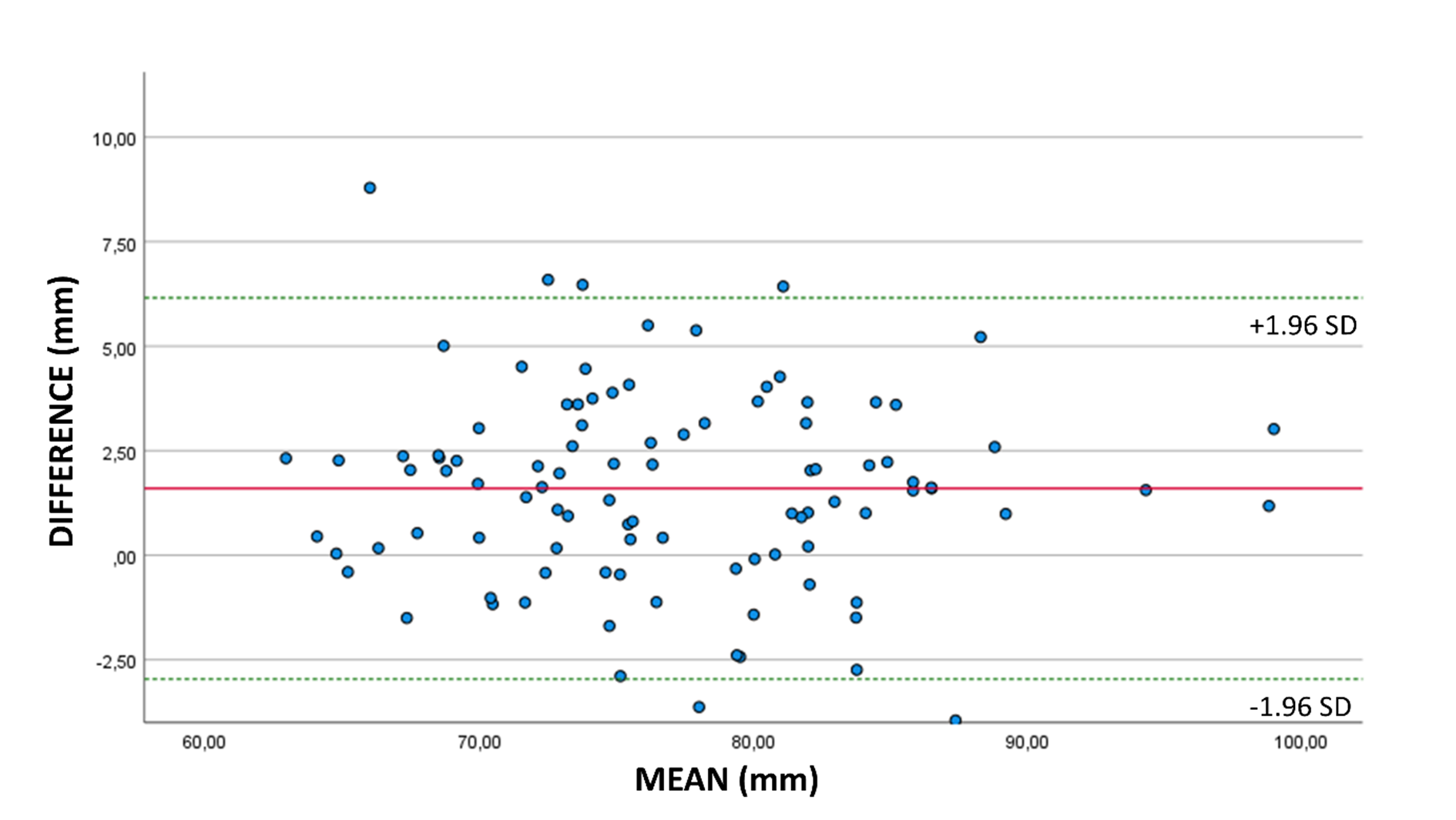

Supplement: Supplementary file 1 — Supplementary Material 1. Bland–Altman plot of agreement between both measurement methods for the aortic annulus perimeter. The red line indicates the mean difference and the dashed green lines the upper and lower limits of agreement (PNG 188 KB) [file 392_2025_2790_Fig4_ESM.png]

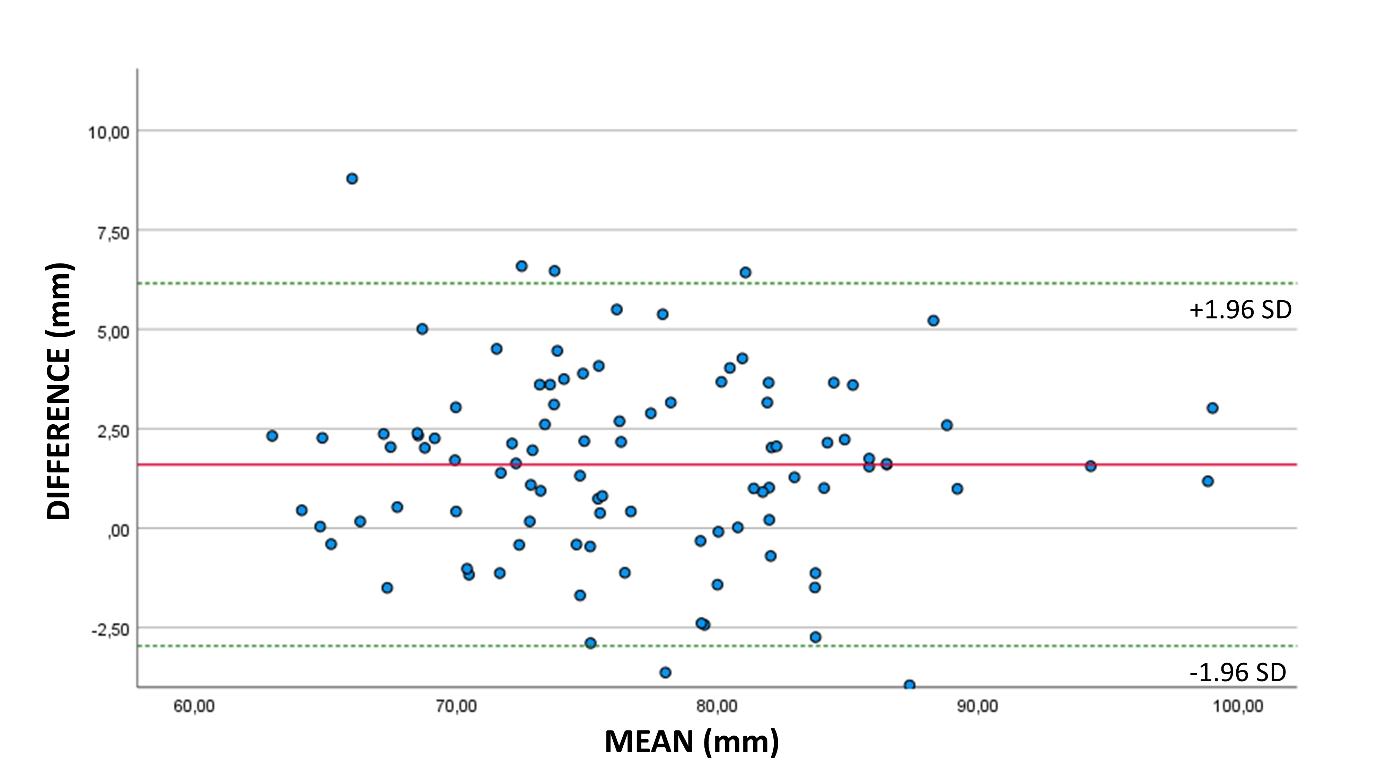

Supplement: Supplementary file 2 — High Resolution Image (TIF 171 KB) [file 392_2025_2790_MOESM1_ESM.tif]

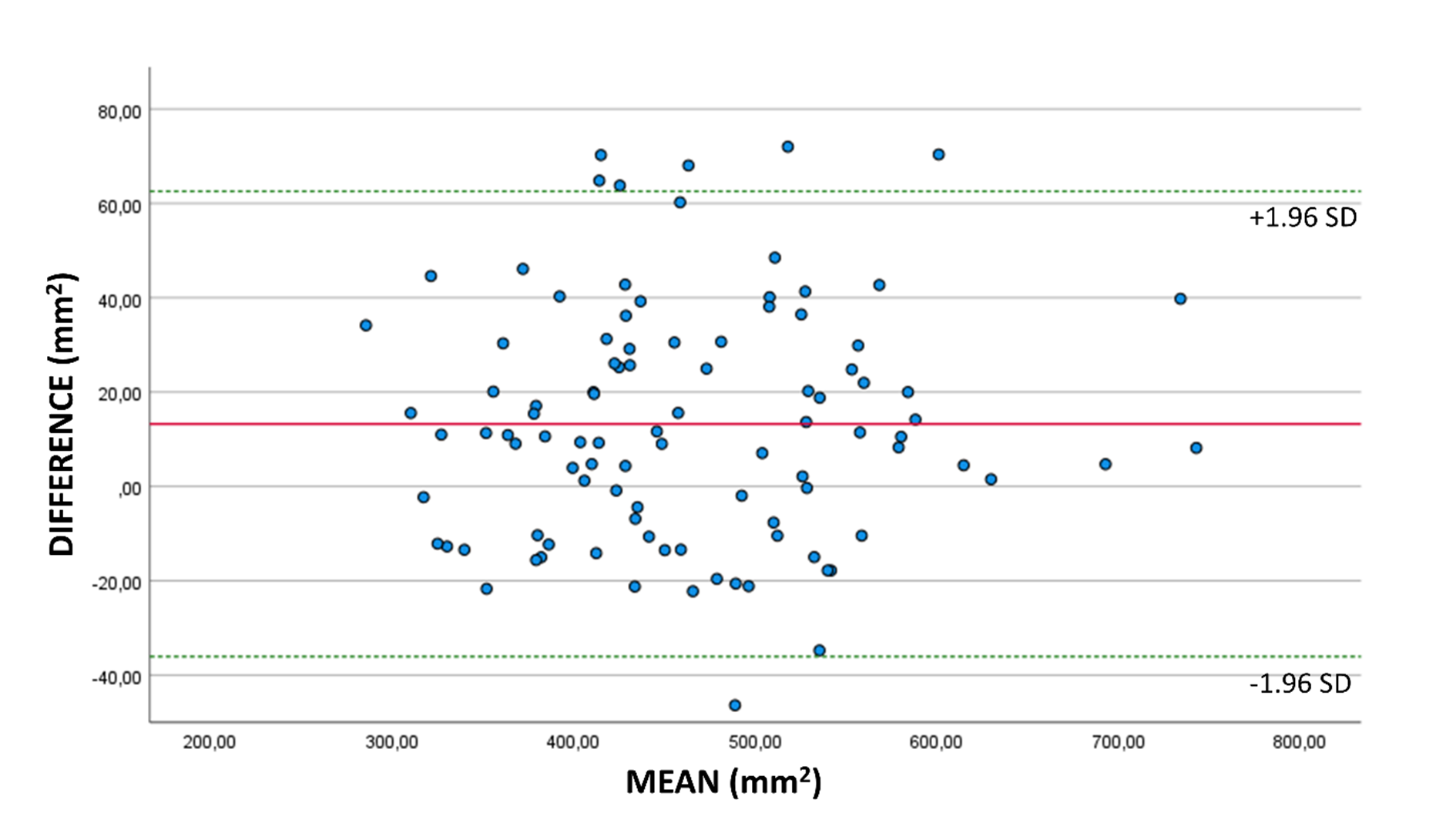

Supplement: Supplementary file 3 — Supplementary Material 2. Bland–Altman plot of agreement between both measurement methods for the aortic annulus area. The red line indicates the mean difference and the dashed green lines the upper and lower limits of agreement (PNG 202 KB ) [file 392_2025_2790_Fig5_ESM.png]

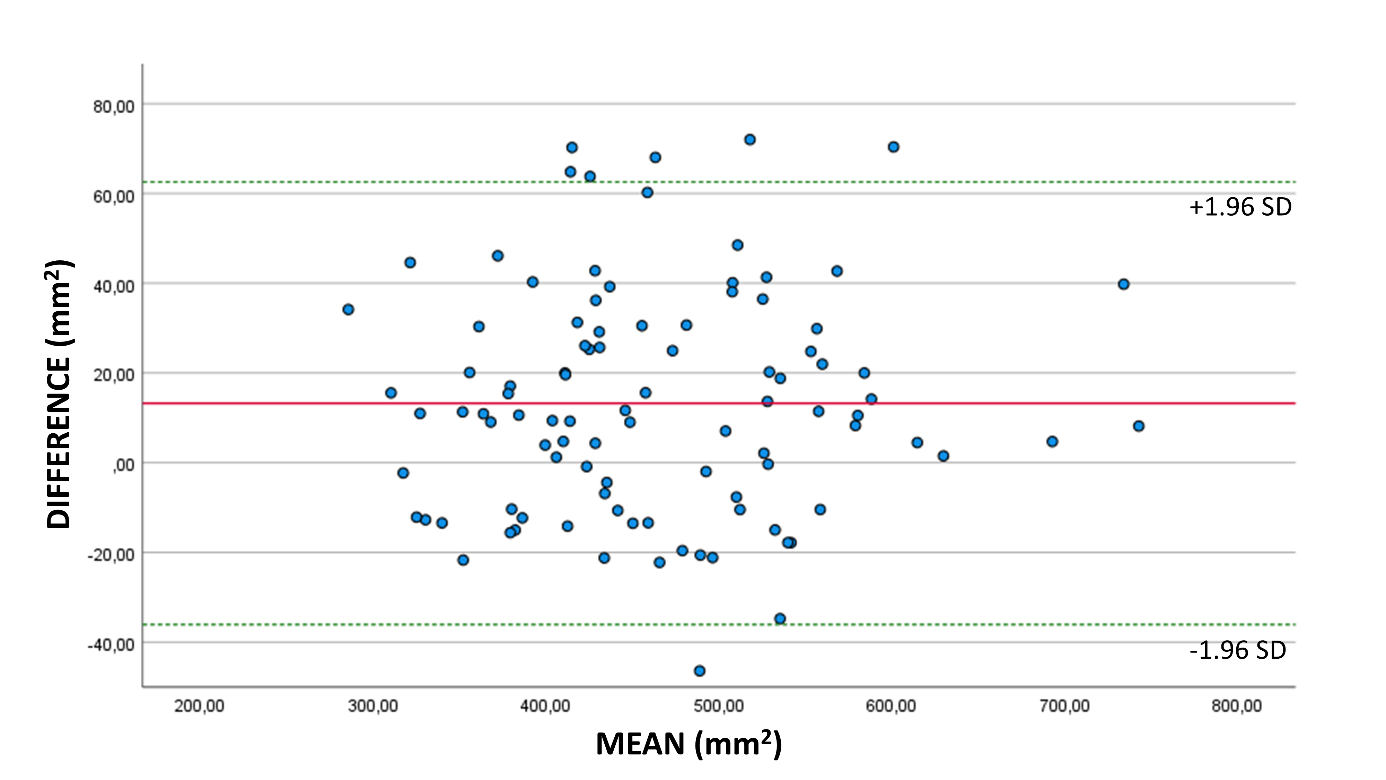

Supplement: Supplementary file 4 — High Resolution Image (TIF 176 KB) [file 392_2025_2790_MOESM2_ESM.tif]

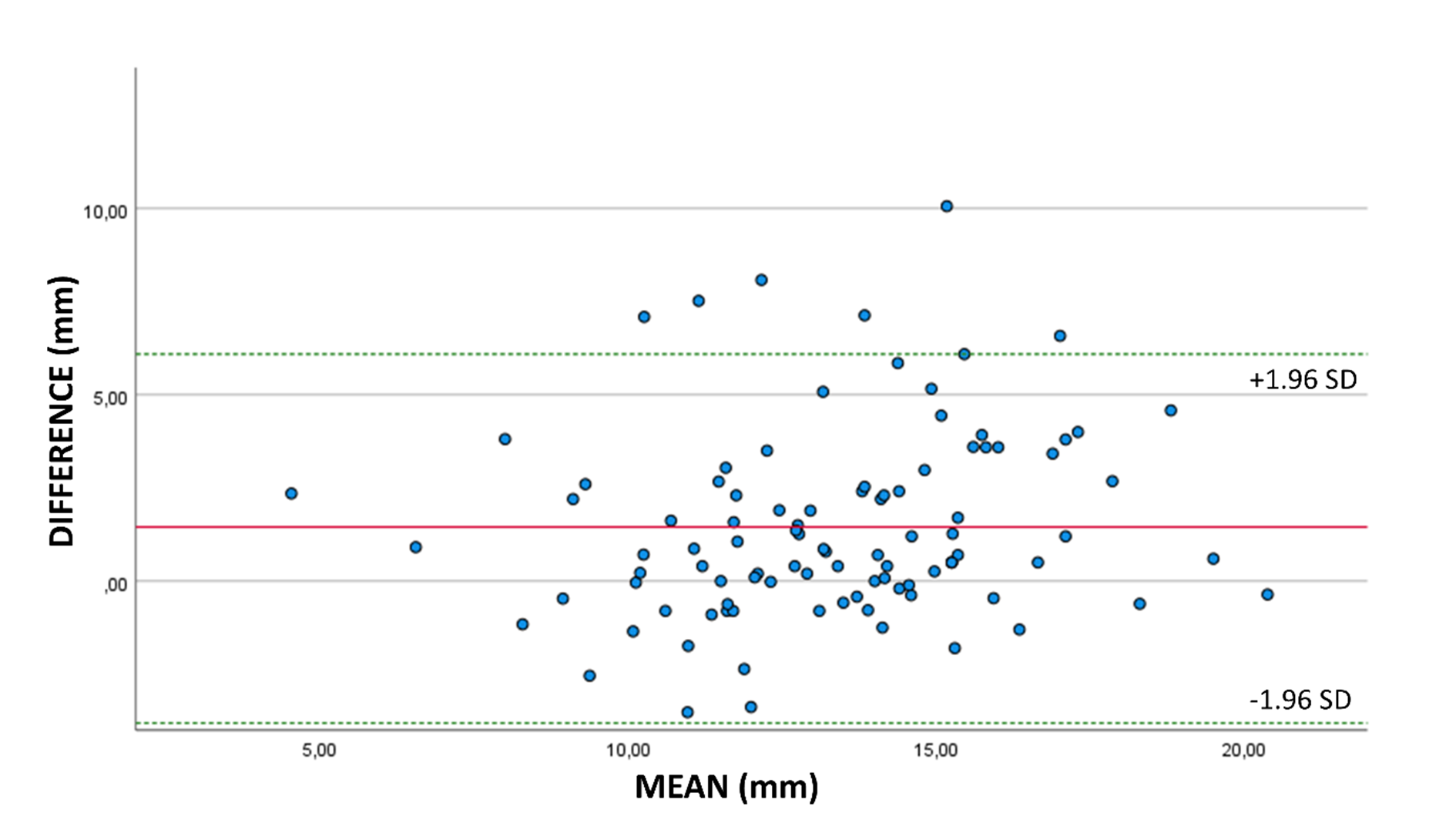

Supplement: Supplementary file 5 — Supplementary Material 3. Bland–Altman plot of agreement between both measurement methods for the distance between the annular plane and the ostium of the left coronary artery. The red line indicates the mean difference and the dashed green lines the upper and lower limits of agreement (PNG 179 KB) [file 392_2025_2790_Fig6_ESM.png]

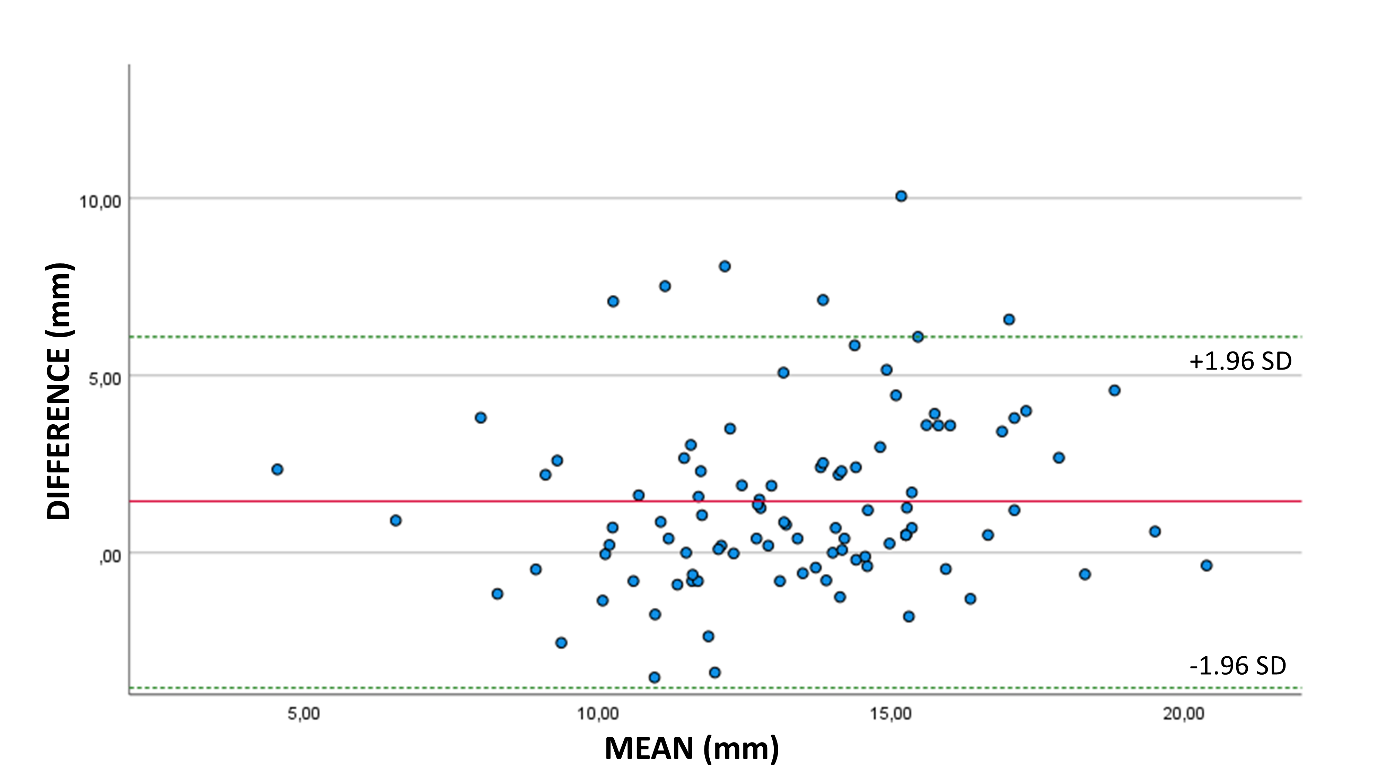

Supplement: Supplementary file 6 — High Resolution Image (TIF 165 KB) [file 392_2025_2790_MOESM3_ESM.tif]

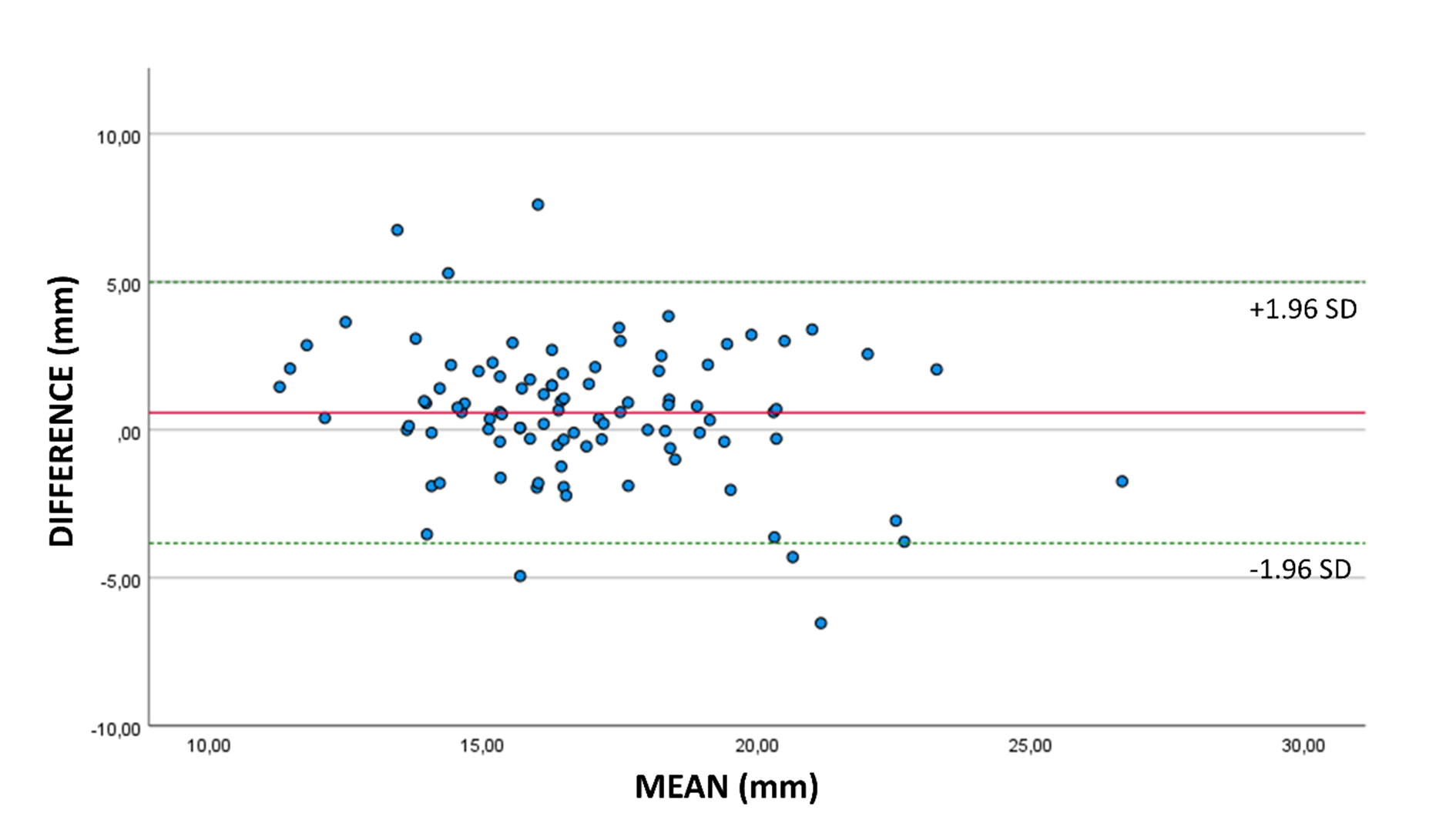

Supplement: Supplementary file 7 — Supplementary Material 4. Bland–Altman plot of agreement between both measurement methods for the distance between the annular plane and the ostium of the right coronary artery. The red line indicates the mean difference and the dashed green lines the upper and lower limits of agreement (PNG 181 KB ) [file 392_2025_2790_Fig7_ESM.png]

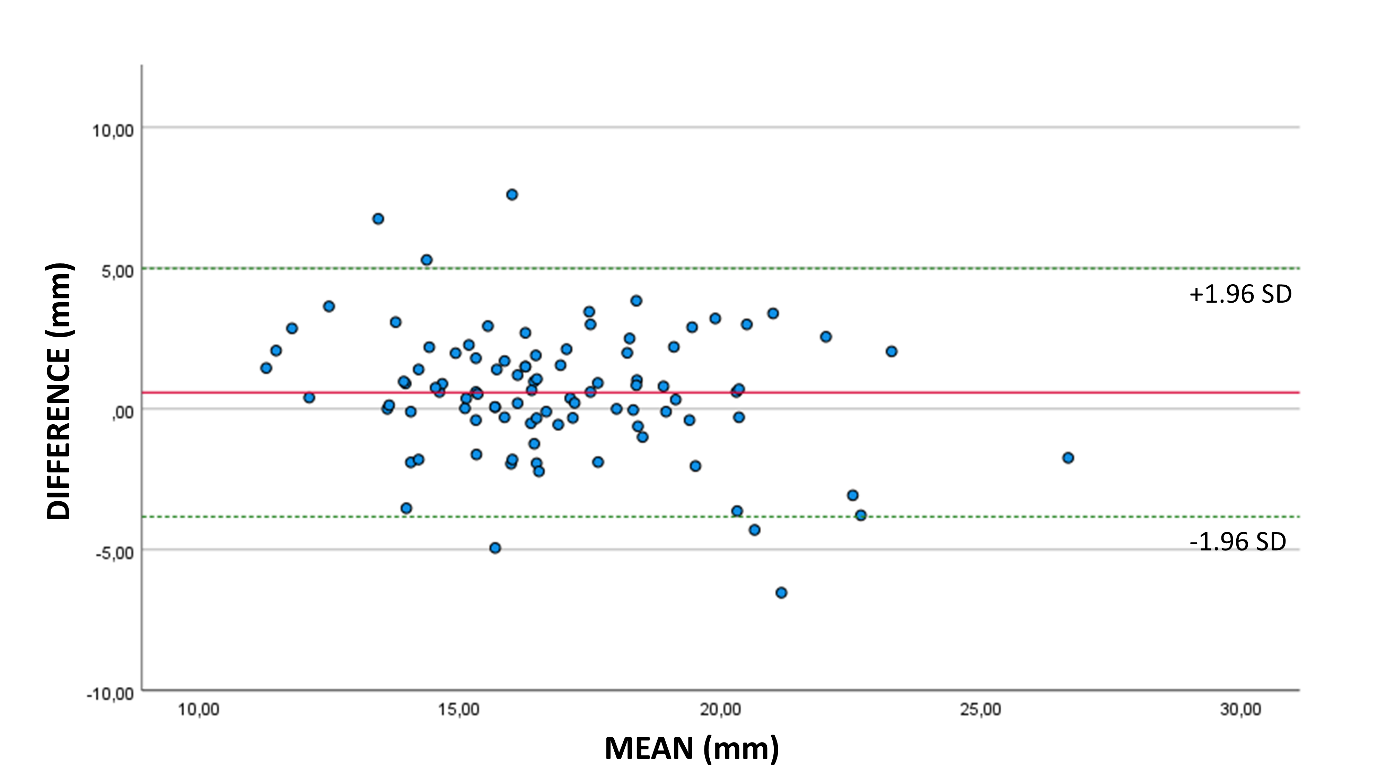

Supplement: Supplementary file 8 — High Resolution Image (TIF 166 KB) [file 392_2025_2790_MOESM4_ESM.tif]
